# Using a verbal prompt to increase protein consumption in a hospital setting: a field study

**DOI:** 10.1186/s12966-015-0271-8

**Published:** 2015-09-17

**Authors:** Lotte D. T. van der Zanden, Harmen van Essen, Ellen van Kleef, René A. de Wijk, Hans C. M. van Trijp

**Affiliations:** Marketing and Consumer Behaviour Group, Department of Social Sciences, Wageningen University, Wageningen, The Netherlands; Food and Biobased Research, Consumer Science & Intelligent Systems, Wageningen University, Wageningen, The Netherlands

**Keywords:** Protein, Prompt, Hospital, Field study, Praise

## Abstract

**Background:**

Sufficient protein intake among hospitalized patients may contribute to faster recovery and a decrease in healthcare costs. Nevertheless, hospitalized patients are often found to consume too little protein. This field study explored the success of a small, inexpensive intervention adapted from the marketing literature, to encourage protein consumption among hospitalized patients.

**Methods:**

The study was performed at a hospital where patients order food by calling to the meal service. The intervention consisted of a verbal prompt: “Would you like some [target product] with that?”, which was presented to patients by trained telephone operators, after patients finished ordering their lunch. Target products were two foods rich in protein; fruit quark and yoghurt drink. For half of the patients, the verbal prompt was preceded by verbal praise on their lunch order, which was aimed to increase compliance with the verbal prompt.

**Results:**

Three hundred and fifteen hospitalized patients, aged 18–87 years took part in the study. Verbal prompts significantly increased ordering of the target products nearly sevenfold (from ordering by 6.5 % of patients to 45.2 % of patients). Protein content of ordered lunch and all food orders of the day combined showed a trend, with orders of patients receiving only a verbal prompt or a verbal prompt and verbal praise containing a larger amount of protein than lunch orders of patients in the control condition. At an individual level, protein content of ordered food increased significantly, reaching the 25–30 g of protein per main meal recommended by dieticians of the hospital. Verbal praise did not increase compliance with the verbal prompt. Patients consumed most or all of the target product and verbal prompts were not perceived to be obtrusive.

**Conclusions:**

Although changing eating patterns is challenging, this study shows that simple interventions such as verbal prompts may be useful tools for health professionals to stimulate healthy food consumption among patients during hospitalization.

## Background

Proteins are large, complex molecules that play a role in various bodily processes, such as supporting the immune system, transporting molecules and speeding up biochemical processes [1, 2]. As a result, bodily protein is gradually expended and needs to be replenished by means of food consumption [2]. Sufficient protein intake is especially important when people get ill, because expenditure of protein increases considerably during sickness and recovery [3]. Studies have shown that patients can benefit in various ways from consuming enough protein. Patients who increase their protein consumption rehabilitate faster from fractures [4], have a reduced risk of developing pressure ulcers (i.e. bedsores) [5], have a lower chance of being readmitted to the hospital [6] and lose less weight during hospital stay [7].

Ensuring sufficient protein intake among hospitalized patients may thus contribute to faster recovery and a decrease in healthcare costs. Nevertheless, hospitalized patients are often found to consume too little protein [8]. Motivating protein consumption among patients is challenging, because protein-rich foods are relatively difficult to chew and swallow [9], instigate aversion more easily than foods rich in carbohydrates [10], and even tend to reduce appetite [11].

### Interventions

Although most consumers are aware that they have to eat certain foods and avoid others, this awareness often does not translate into actually eating a healthy diet. Consumers tend to believe that healthy foods are less tasty than unhealthy foods [12], making it difficult for health professionals to stimulate healthy food consumption among their clients, even among clients whose current health status would directly benefit from better food choices [13, 14]. Increasing the attractiveness of choosing and eating healthy foods may help health professionals to inspire dietary changes among their patients.

With its vast background of research on affecting consumer choice, the field of marketing can provide useful input into dietary interventions. Although marketing techniques were traditionally used to increase product sales, they can also be applied and are increasingly applied in the best interest of the consumer. Research within this field of “social marketing” shows that relatively small and inexpensive changes in the choice environment can already motivate people to make better choices [15], by exploiting the fact that the majority of our everyday choices are made without much deliberation [16]. To stimulate healthy food choices in a canteen, for example, one could place healthy foods in more easy-to-reach places [17] or complement healthy foods with appealing descriptions such as “home-made” [18]. Interventions such as these could also be used to encourage adequate protein consumption among hospitalized patients.

Adequate intake of nutrients, such as protein, is the result of multiple, individual food choices made over time. Ideally, an intervention aimed at increasing protein consumption among hospitalized patients would increase protein intake within meals, without eliciting compensation behaviours at the same or a later point in time. The present study tried to take this into account by implementing an intervention right after patients completed their orders, leaving little room for immediate compensation. In addition, food orders made during the rest of the day were examined for potential compensation effects.

### Verbal prompt

Implementing an intervention right after consumers make their food choices provides ideal circumstances for a small and inexpensive technique adapted from the marketing literature: the verbal prompt. A verbal prompt is a product suggestion given in a question format, such as “Would you like a side of salad with your meal?”, aimed to motivate consumers to purchase a product that broadly complements what they have already ordered or purchased [19]. Verbal prompts provide consumers with a mandatory choice that requires an active affirmation or rejection of the product suggestion. In forced yes/no questions like these, receivers tend to display an acquiescence (i.e. affirmation) bias, responding more often with ‘yes’ than with ‘no’ [20].

This affirmation bias in response to verbal prompts has various underpinnings, among which two main mechanisms can be identified. Firstly, due to a limited cognitive capacity, people are constrained in the extent to which they can deliberate on their actions and decisions. As a result, consumers often rely on habits or heuristics and make decisions without much thought [16]. Secondly, people are motivated to act in a socially desirable way in order to convey a favourable image of themselves [21] and tend to cooperate or reciprocate even in one-shot interactions (i.e. with no expected future exchanges) [22].

Verbal prompts are commonly used in retail settings such as restaurants (e.g. “would you like another drink?”), gas stations (e.g. “would you like a coffee?”) and fast food chains (e.g. “would you like fries with that?”) to increase the sales of these target products. More recently, verbal prompts have been used in interventions to promote desirable consumption behaviours, such as reducing portion sizes in fast-food restaurants [23], increasing fruit and vegetable consumption in schools [24] and increasing consumption of healthy side dishes at a cafeteria [25]. The current study aimed to stimulate protein consumption among hospitalized patients using a verbal prompt.

Given that the most crucial component in the success of verbal prompt interventions is consumers’ compliance with the verbal prompt (i.e. ordering the product suggestion), this study complemented a verbal prompts intervention with a technique aimed to increase compliance: verbal praise. Verbal praise has been shown to increase compliance across various contexts, ranging from direct requests [26] and purchase of merchandise [27] to tipping behaviour [28]. The compliance-enhancing effects of verbal praise are thought to be based on both interpersonal processes, such as liking of, or reciprocity towards the praise-giver [29], and intrapersonal processes, such as self-enhancement [30] and an increased motivation to perform the praised behaviour [31]. We expect that verbal praise, when preceding a verbal prompt, will increase compliance with the verbal prompt and increase the success of a verbal prompt intervention.

## Methods

### Setting

The field study took place at a 600-bed hospital in the Netherlands, which covers an area of 260,000 residents and has a mean annual admission rate of more than 21,000 patients. The hospital makes use of Sodexo’s At Your Request meal-service program [32], which is used in over 350 hospitals worldwide. In this program, patients order meals, drinks and snacks from a restaurant style menu by calling the meal service, after which the order is freshly prepared and delivered to their room within 45 min. On a daily basis, about 400 food orders are placed in the hospital where this study took place.

Lunch and breakfast orders were identified by dieticians of the hospital as containing too little protein and were thus both suitable targets for our intervention. However, breakfast could be ordered both in the evening and morning whereas lunch could only be ordered during the same day. This difference in the time frame of ordering made the lunch order a more practical target and was therefore selected as the focus of our intervention.

### Design

The food ordering system was used as a basis for our interventions (i.e. the verbal prompt and verbal praise), which were given to the patients by five female telephone operators of the meal service. Depending on the condition, operators provided patients with only the verbal prompt (verbal prompt condition), both the praise and the prompt (praise-then-prompt condition) or none of the interventions (control condition).

### Subjects

A total of 315 patients took part in the study. Patients automatically took part when they 1) were aged 18 or older, 2) did not have a protein-restricted diet, 3) personally called to place an order, and 4) placed an order for lunch. Participants were 18–87 years old (M = 60.6, SD = 17.8) and the total dataset consisted of 46.0 % males and 54.0 % females. Patients were not notified of their participation in the experiment, because we could not predict which patients would call the telephone operator that took part in the experiment. Informing the patients at the start of the telephone conversation was not desirable either, given that this would likely influence the results of the interventions. This and all other procedures were approved by the research ethics committee (BCWO) of Hospital Gelderse Vallei.

### Procedure

The study took place before lunchtime on fourteen weekdays of four consecutive weeks, and each of the three conditions was carried out on at least four different days of the week. Each telephone operator carried out all three conditions on different days of the week, but in the same order. The control condition was always carried out on the first of three days (i.e. no intervention), the verbal prompt condition on the second day and the praise-then-prompt condition on the third day. This way, training for the final condition was built up in a stepwise fashion, making the final condition easier for the telephone operators to execute. A pilot study was used to assess the clarity of instructions for the telephone operators and to identify potential practical issues with collecting the data.

Starting every experiment day, the telephone operator that was scheduled for that day was instructed on the condition that she would execute. The condition was practiced with the researcher, if necessary, and when telephone operators could give the praise and prompt according to the instructions, the experiment was started. During the control condition and the experiment, the telephone operator was seated at a desk with a computer in which she entered the lunch orders. One of two researchers was seated next to her and recorded information on a checklist that would not be recorded in the computer of the telephone operator (e.g. whether the praise and prompt were given according to the instructions). In the control condition, telephone operators were asked to answer calls as usual. The data from this condition were used as a baseline. In the verbal prompt condition, telephone operators answered calls as usual, but ended the call with a verbal prompt. In the praise-then-prompt condition, telephone operators answered calls as usual, but ended the call with praise on the order of the patient and a verbal prompt. After lunch time, two researchers visited a subset of the patients (*N* = 128, 40.6 % of the total dataset) in their rooms with a short questionnaire about their lunch order. All patients who were awake and in their rooms agreed to fill out the questionnaire after giving written informed consent.

### Interventions

Verbal prompts were given by telephone operators right after patients finished their order and consisted of the following construction: “Would you like some [target product] with that?”. Two dairy products were selected as targets for the verbal prompt in consultation with the dieticians of the hospital: fruit quark (i.e. a dessert made from fresh cheese with fruit) and yoghurt drink. These products contain a mean of 7.1 g of protein per portion but are also smooth in texture. As a result, they may be more easy to swallow and less satiating than other protein-rich foods [33], overcoming some of the physical barriers to protein consumption. Fruit quark was used as the main target for the prompt (used in 68.2 % of cases), but when patients had already ordered fruit quark, or another dessert, they were prompted with yoghurt drink (used in 31.8 % of cases).

**Fig. 1:**
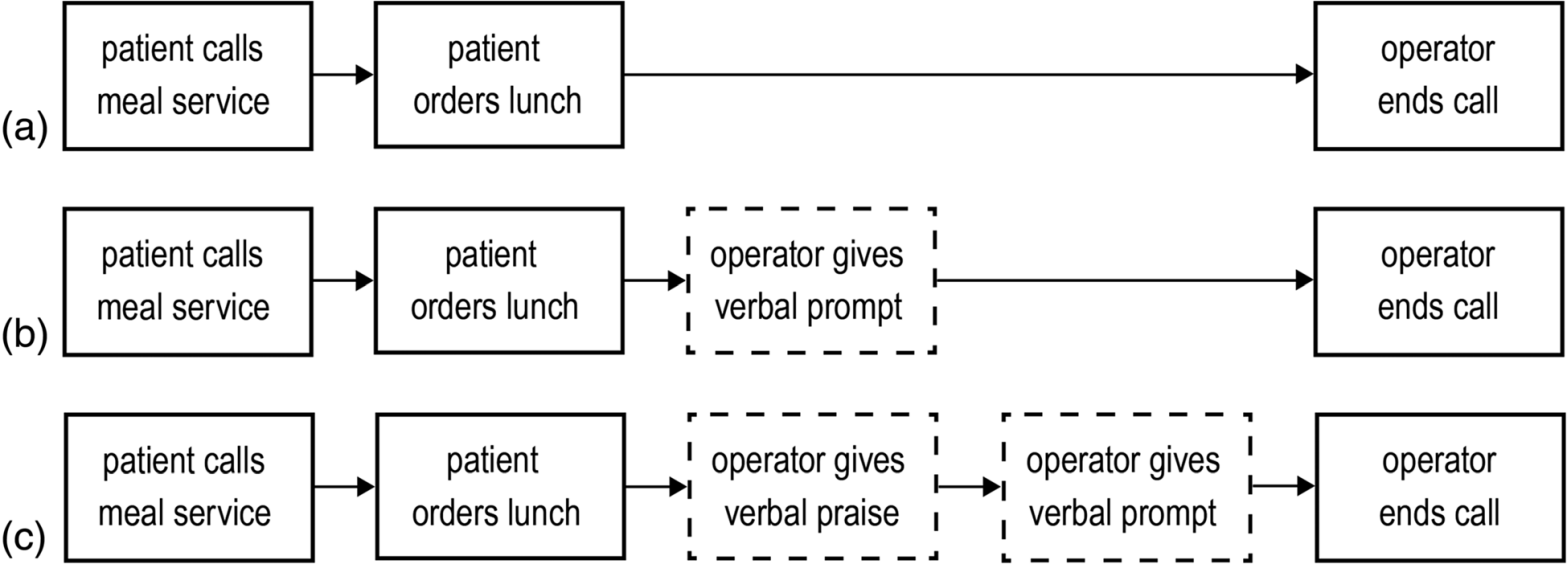
Overview of the meal ordering procedure for each of the three conditions. The control condition (**a**) served as a baseline, during which patients were not exposed to any of the interventions. In the verbal prompt condition (**b**), patients received only the verbal prompt and in the praise-then-prompt condition (**c**), patients received verbal praise, followed by the verbal prompt

Verbal praise consisted of the following construction: “Good that you ordered [food product]”. If a consumer perceives a clear ulterior motive for flattery by a salesperson, the initial positive reaction to praise is replaced by a less favourable one [34]. Therefore, praise was given right before patients were presented with a verbal prompt, reducing the possibilities for patients to deliberate on the motive for giving the praise (Fig. 1) [35]. Moreover, the food product referred to in the praise was based on a concrete food choice that patients made for their lunch order, such that praise was personalised, and thus varied from patient to patient. This was done to reduce perceived insincerity of the praise. In addition, telephone operators were instructed to refer in their praise to a specific product in patients’ orders (e.g. “good that you ordered brown bread”) rather than providing patients with more general praise (e.g. “you made a good choice”). When the lunch order of a patient was too unhealthy (*N* = 4) or small (*N* = 15) to serve as a reasonable basis for praise (e.g. when a patient ordered snack foods or just a cup of coffee), telephone operators were instructed to not provide praise and these patients were not included in the study. In the data analysis, we explored whether excluding these patients distorted the sample of patients in the praise-then-prompt condition in terms of meal size or healthiness, by looking at the salt, fat and caloric content of their food orders.

### Checklist and questionnaire

On a checklist, the researchers recorded who was calling to give an order (i.e. patient vs. nurse/family/friends), whether the verbal prompt and praise were given, which target product was used for the prompt and the praise, and whether the verbal prompt and praise were given following the instructions. In addition, the checklist recorded the information necessary to visit patients with a questionnaire (i.e. patient name, hospital wing, room and bed number).

Verbal prompts are sometimes perceived to be obtrusive or pushy [36] and may cause resistance or even counterproductive effects (e.g. increasing unhealthy food consumption) [37, 38]. However, when care is taken, perceived obtrusiveness can be avoided [25]. The questionnaire was used to assess the extent to which patients perceived the verbal prompt to be obtrusive. Patients were asked to rate their agreement with 4 statements: “The telephone operator was pushy suggesting the dish”, “I found it hard to say ‘no’ to the telephone operator”, “I felt obligated to comply to the suggestion of the telephone operator”, and “Receiving a suggestion from the telephone operator was annoying”. Ratings were made on a 5-point Likert scale ranging from 1 = “strongly disagree” to 5 = “strongly agree”. Items were adapted from van Kleef, van den Broek and van Trijp [25].

Given that *ordering* the target product following the verbal prompt does not necessarily mean that patients will actually *consume* it [8], the paper questionnaire also assessed to what extent patients consumed the target product. If patients had ordered fruit quark or yoghurt drink, irrespective of whether they received a verbal prompt, they were asked to what extent they had consumed these products (using a 5-point Likert scale with the response options 1 = “ate none of it”, 2 = “ate some of it”, 3 = “ate half of it”, 4 = “ate most of it”, and 5 = “ate all of it”).

Lastly, the questionnaire contained a collection of questions added as control variables. One set of questions was used to assess patients’ reasons for (not) ordering the target product. Patients could choose from multiple response options (e.g. It is (not) good for me, I automatically responded, I did (not) feel like eating it) or write down any other reason. Patients who did not receive the verbal prompt were instructed to skip this set of questions. A second set of questions was used to assess whether the verbal prompt and verbal praise had any negative side effects on perceptions of the telephone operator and telephone conversation by the patients. Three items were used: “The telephone operator was helpful”, “The telephone operator was friendly”, and “The telephone conversation was pleasant”. Ratings were made on a 5-point Likert scale ranging from 1 = “strongly disagree” to 5 = “strongly agree”. All patients were asked to fill out this set of questions.

### Outcome and background measures

Three measures were used to assess the success of the interventions: ordering of the target product for lunch and protein content of the ordered lunch. In addition, the caloric content of the ordered lunch was used as descriptive variable. *Ordering of the target product* for lunch was operationalized as a binary variable indicating whether or not patients ordered fruit quark or yoghurt drink, irrespective of whether or not they had received the prompt. *Protein and caloric content of the ordered lunch* were operationalized as the number of grams of protein and the number of kilocalories in ordered food, respectively.

As protein supplementation may cause patients to eat less during subsequent meals [39], we also assessed the number of grams of protein and kilocalories in the breakfast and dinner orders, and across all food orders of the day (i.e. daily protein and caloric content). Given that about half of the patients did not remain in the hospital for the whole day, daily protein and caloric content were only analysed for patients that ordered breakfast, lunch and dinner.

All data for background measures, protein and caloric content were retrieved from the hospital database. In this database, nutritional values of processed foods such as bread, cheese and soup were based on the product specifications provided by the producer. Nutritional values of unprocessed foods such as fruit, vegetables and herbs, as well as processed products for which product specifications were not yet available, were adapted from the NEVO table (i.e. Dutch Nutrient database) [40].

### Data preparation

Patients were excluded from the data analysis when they did not receive the prompt in the instructed format (e.g. telephone operators used both target products in one verbal prompt) (*N* = 16, 16.2 % of patients in the verbal prompt condition) or when they did not receive the praise in the instructed format (e.g. telephone operators did not refer to a specific product in the patient’s lunch order) (*N* = 37, 34.9 % of patients in the praise-then-prompt condition). Six patients were excluded as outliers because the protein or caloric content of their food orders exceeded 3 or more standard deviations from the mean (1.9 % of the total dataset). Questionnaire data were discarded when patients provided reasons for (not) ordering that were inconsistent with their actual ordering pattern (e.g. patients did not comply with the verbal prompt, but indicated that they “did not dare to say ‘no’ to the telephone operator”) (*N* = 5, 3.9 % of all questionnaires). For 47 patients who participated in the study more than once, all data collected after the first participation were excluded from the data analysis (14.9 % of the total dataset). The remaining dataset consisted of 102 filled-out questionnaires and 208 lunch orders made by 208 patients, of which 93 were in the control condition (44.7 %), 62 in the verbal prompt condition (29.8 %) and 53 in the praise-then-prompt condition (25.5 %). This sample of patients will be referred to as the ‘total sample’ from here on.

### Data analysis

Data were analysed using SPSS version 22.0 (IBM Inc., Chicago, IL). Continuous data (e.g. protein and caloric content of orders) were analysed using (Multivariate) Analyses of Variance ((M) ANOVA’s), categorical data (e.g. ordering of the target product) were analysed using non-parametric Chi-Square tests.

## Results

### Ordering of the target products

Descriptive statistics indicated that, in the total sample of patients, target products (i.e. fruit quark or yoghurt drink) showed up in lunch orders for 6.5 % of patients in the control condition, 45.2 % of patients in the prompt condition and 45.3 % of cases in the praise-then-prompt condition. A non-parametric Chi-Square tests with condition as the independent variable and ordering of the target (i.e. “yes” versus “no”) as the dependent variable showed a main effect of condition on ordering of the target product, *χ*^2^ (2, *N* = 208) = 38.426, *p* < .001. Bonferroni-corrected pairwise comparisons between the conditions (with a test value of .05/3 = .016) indicated that the target product was ordered significantly less often in the control condition than in the prompt condition and the praise-then-prompt condition (both *p* values < .001). The verbal prompt and praise-then-prompt condition did not differ significantly from each other, *p* = .990.

### Protein content of the ordered lunch

Lunch orders of the total sample of patients contained a mean of 25.4 g of protein and 571.8 kcal. An ANOVA with condition as the independent variable and protein content of the lunch order as dependent variable was used to test whether condition had an effect on protein content of the lunch order of the total sample of patients (i.e. including those who did not comply with the prompt). Age, diet, gender and caloric content of food orders were accounted for. The analysis showed a trend, with lunch orders of patients in the verbal prompt and praise-then-prompt conditions containing a larger amount of protein than lunch orders of patients in the control condition (*p* = .077, eta^2^ = .027) (see Table 1). When excluding patients who did not comply with the verbal prompt (*N* = 63), the main effect of condition on protein content of the lunch order was significant (*p* < .001, eta^2^ = .168) (see Table 1).

**Table 1:** Protein content of the lunch order per condition

	Control			Prompt			Praise-then-prompt			
	*N*	*M*	*SD*	*N*	*M*	*SD*	*N*	*M*	*SD*	*p-value*
Total sample (*N* = 208)	93	24.0	10.8	62	26.3	11.0	53	27.0	11.2	.077
Total sample excl. non-compliers (*N* = 145) ^a^	93	24.0	10.8	28	30.3	10.5	24	30.7	9.8	< .001

^a^ total sample excluding patients who did not comply with the verbal prompt

### Daily protein and caloric content

Combined over the whole day, food orders of the total sample of patients contained a mean of 77.1 g of protein and 1712.8 kcal. An ANOVA with condition as the independent variable and protein content of all orders of the day combined as dependent variable was used to test whether condition contributed significantly to daily protein content of ordered food. Age, diet, gender and caloric content of food orders were accounted for. A total of 99 patients (i.e. including those who did not comply with the prompt) ordered breakfast, lunch and dinner (47.6 % of the total sample), and were included in this analysis. The analysis showed a trend, with food orders of patients in the verbal prompt and praise-then-prompt conditions containing a larger amount of protein than lunch orders of patients in the control condition (*p* = .095, eta^2^ = .056) (see Table 2). When excluding patients in this group who did not comply with the verbal prompt (*N* = 27), the main effect of condition on protein content of food orders was significant (*p* = .025, eta^2^ = .121) (see Table 2).

**Table 2:** Protein content of the daily order per condition

	Control			Prompt			Praise-then-prompt			
	*N*	*M*	*SD*	*N*	*M*	*SD*	*N*	*M*	*SD*	*p-value*
All-meal patients ^a^ (*N* = 99)	42	73.6	31.5	30	78.2	27.3	27	81.4	26.1	.095
All-meal patients excl. non-compliers (*N* = 72)	42	73.6	31.5	15	82.2	33.2	15	84.3	23.1	.025

^a^ patients who ordered breakfast, lunch and dinner

To test whether patients who complied with the verbal prompt did this to compensate for a breakfast that was small, or low in protein, a MANOVA was performed with ordering of the target product as the independent variable, and protein and caloric content of breakfast as the dependent variables. Age, diet and gender were accounted for. A total of 144 patients ordered breakfast. There was no significant difference in protein or caloric content of breakfast between patients who complied with, or did not comply with the verbal prompt (both *p* values > .100). This analysis was repeated for the dinner order, to test whether patients who complied with the verbal prompt compensated for this by ordering a small or low-protein dinner later. A total of 127 patients ordered dinner. There was no significant difference in protein or caloric content of dinner between patients who complied with, or did not comply with the verbal prompt (both *p* values > .100) (see Table 3).

**Table 3:** Breakfast and dinner of patients who complied with, or did not comply with the verbal prompt

		Compliers			Non-compliers			
		*N*	*M*	*SD*	*N*	*M*	*SD*	*p-value*
Breakfast (*N* = 144)	Protein content	38	19.7	10.9	106	19.3	13.0	.709
	Caloric content	38	493.1	229.9	106	473.3	242.1	.675
Dinner (*N* = 127)	Protein content	40	29.2	8.9	87	26.0	10.1	.266
	Caloric content	40	610.2	216.6	87	554.1	198.1	.157

To test whether the sample of patients in the praise-then-prompt condition was distorted in terms of meal size or healthiness (due to exclusion of patients with meals that were an inappropriate basis for praise), a MANOVA was performed on the total sample of patients with condition as the independent variable, and fat content, salt content and carbohydrate content of all orders of the day combined as the dependent variables. Age, diet, gender, ordering of the target product and caloric and protein content of food orders were accounted for. The analysis showed no significant differences between the conditions in terms of fat, salt or carbohydrate content (all *p*-values > .100).

### Reasons for ordering the target product

Data from the questionnaire were used to gain insight into reasons for (not) ordering the target product. The questionnaire was filled out by 49.1 % (*N* = 102) of the total sample of patients and Chi-Square tests and an ANOVA indicated that respondents were representative of the total sample in terms of gender, condition and how often the target product was ordered, but not in terms of age. Respondents to the questionnaire were significantly older (M = 62.2, SD = 18.7) than patients in the total sample (M = 57.0, SD = 18.7) (*p* = .034, eta^2^ = .022).

Descriptive statistics showed that, among all respondents to the questionnaire who received the verbal prompt (*N* = 62), the expected taste of the target products was mentioned most often as a reason (not) to order the target product (mentioned by 58.1 % of the respondents). To look into differences in the reasons reported by respondents who did or did not comply with the verbal prompt, multiple non-parametric Chi-Square tests were performed, with ordering of the target as the independent variable and reporting of reasons for (not) ordering the target product as dependent variables (see Table 4). The two groups differed significantly in terms of reporting that the target product, suggested by the telephone operator, “was (not) a useful suggestion” (*p* = .013) and marginally significantly in that they “automatically responded” to the suggestion (*p* = .058). Patients who complied with the verbal prompt reported both reasons more often than patients who did not comply with the verbal prompt.

**Table 4:** Percentage of patients who reported the reason for (not) ordering the target product

	Compliers (*N *= 27)	Non-compliers (*N* = 35)	*p-value*
I do (n’t) like the taste	66.7 %	51.4 %	.301
It is (not) good for me	40.7 %	31.4 %	.593
I did (not) feel like eating it	40.7 %	34.3 %	.791
It was (not) a useful suggestion	48.1 %	17.1 %	.013
I automatically responded	33.3 %	11.4 %	.058
I ordered enough already	n.a.	11.4 %	.125
I did not dare to say ‘no’	3.7 %	n.a.	.435

There was no significant difference between patients who did or did not order the target product in reporting of the reason “I did not dare to say ‘no’ to the telephone operator” (*p* = .435), suggesting that obtrusiveness was not a reason for patients to comply with the verbal prompt. In line with this finding, the four items used to measure perceived obtrusiveness received low mean ratings, ranging from M = 1.3 to M = 2.4 (rated on a scale from 1 = “strongly disagree” to 5 = “strongly agree”) and did not differ significantly between those who complied with, or did not comply with the verbal prompt, all *p* values ≥ .331.

The questionnaire also contained a control question aimed to assess if patients actually consumed the target product once they ordered it. Descriptive analyses of this data showed that 65.0 % of the respondents who ordered the target product reported eating “most” or “all” of the target product (25 out of 33 patients). Only 1 patient reported not having consumed the target product after ordering it.

### Background characteristics

To gain some insight into potential side-effects of the verbal prompt and verbal praise, the friendliness and helpfulness of the telephone operators and the pleasantness of the conversation as evaluated in the questionnaire by patients were compared among the three conditions. ANOVA’s indicated that these variables did not differ significantly between the conditions, all *p* values ≥ .109.

Demographic characteristics of patients in the total sample were explored to gain some insight into who did (*N* = 52) and who did not order the target product (*N* = 156). Characteristics with nominal values (i.e. gender and diet (e.g. low-sodium or diabetic diet)) were analyzed using non-parametric Chi-Square tests and characteristics with continuous values (i.e. age) were analyzed using ANOVA’s. The analyses showed that patients who did and did not order the target product did not differ from each other in terms of age, diet or gender, all *p* values ≥ .485.

## Discussion

In this field study, a verbal prompt was used to stimulate protein consumption among hospitalized patients. Compliance to the verbal prompt was considerable: the intervention increased the ordering of two protein-rich target products nearly sevenfold. In addition, protein content of ordered lunch and all food orders of the day combined showed a trend, with orders of patients receiving only a verbal prompt or a verbal prompt and verbal praise containing a larger amount of protein than lunch orders of patients in the control condition. At an individual level, protein content of ordered food increased significantly, reaching the 25–30 g of protein per main meal recommended by dieticians of the hospital. These results show that small, inexpensive interventions can have large effects on food choice and may, consequently, contribute to improving public health and decreasing healthcare costs.

Our study replicates earlier research showing that marketing techniques, like verbal prompts, can also be applied in the best interest of the consumer [23–25], although studies have varied in success. Compliance with the verbal prompt in these studies ranged from 2 to 33 %, depending on the environment (e.g. fast-food restaurant vs. school canteen), target product (e.g. fruit vs. pancakes) and other contextual characteristics (e.g. face-to-face vs. by telephone). The considerable increase in ordering of target products in the current study may likely be explained by some of the predictors of affirmation bias. Affirmation bias is stronger when consumers are not able or motivated to engage in high levels of cognition in making choices [41]. As we studied hospitalized patients, a large part of our subjects may have had other things on their minds (e.g. pain or fatigue due to medical reasons) than thinking deeply about their food choices, which may have made them particularly susceptible to the affirmation bias. In line with this idea, patients who ordered the target product reported more often that they “automatically responded” to the suggestion than patients who did not order the target product.

Another explanation for the success of the verbal prompt in this field study is that food products could be ordered free of charge. Monetary considerations were thus no issue in complying with the offer and likely increased affirmation. However, patients did not just order the target products and dispose of them, they also consumed the products. The majority of the patients indicated that they consumed most or all of the target products. Similar strong effects were found in a school-based intervention to increase fruit consumption, wherein fruit was provided free of charge [24]. Alternatively, the fact that the product suggestions was given by respectable staff members of the hospital could have been a justification for patients to comply with the verbal prompt [42]. Patients in our study might have, rightfully, felt that the product suggestion was selected carefully by the hospital. Indeed, patients who complied with the prompt reported more often than non-complying patients that they thought the target product was a useful suggestion. The verbal prompt may thus have instigated a product demand, which is traditionally the goal of marketing [43].

Verbal praise did not increase compliance with the verbal prompt, which could suggest that patients did not perceive verbal praise to be genuine. This result would be in line with studies on praise which have shown that praise may backfire when observers perceive it to be insincere [34]. However, verbal praise neither increased nor *decreased* compliance with the verbal prompt and telephone operators in the different conditions were not rated as less or more friendly by patients.

More likely, the verbal prompt itself was already convincing enough to yield a strong affirmation bias among patients. Even so, there was no indication that patients felt forced to order the target products and verbal prompts were not perceived to be obtrusive. Given that telephone operators also faced difficulties in correctly providing verbal praise to patients, it may be warranted to use solely a verbal prompt in motivating patients to increase their consumption of target products.

Across the total sample of patients (i.e. including those who did not comply with the prompt) verbal prompts increased protein content of food orders considerably, but only marginally significantly. Most likely, the effect of the intervention was clouded by variance in the data. Variance in protein content of food orders was large despite accounting for variables such as age and gender, for example, ranging from 1 g to 55 g in the lunch orders. Given that studies have shown that protein supplementation may cause patients to compensate by eating less during other meals [39], we examined the protein content of breakfast and dinner orders of the patients. Analyses showed that patients who complied with the verbal prompt were not compensating for ordering a smaller or low-protein breakfast earlier that day and did not compensate by ordering a smaller or low-protein dinner later.

The meal service program of the hospital at which this study was performed provided an ideal setting for applying a simple intervention that would reach many patients. However, this setting also introduced a limitation to this study. Our intervention did not reach patients who were in the earliest stages of their recovery and asked family or nursing staff to call the meal service for them. Given that these patients could have benefited the most from consuming some additional protein, hospitals could give verbal prompts to family members and nursing staff as well. Alternatively, hospitals may want to train the other staff members such as nurses, dieticians and doctors to use the verbal prompting technique on patients. Training other staff members to use the verbal prompt technique may also be a solution for hospitals where food orders are not made by phone. Given that the verbal prompt is a simple and time-efficient intervention, various types of hospital staff should be able to apply it effectively. Future research should, however, look into the circumstances under which verbal prompts are most effective.

To gain some insights in perceived obtrusiveness of the verbal prompt and the actual consumption of the target products, patients were visited by one of the researchers with a paper questionnaire. Data collected from these items supported our use of verbal prompts to increase protein consumption, but conclusions based on these data should be drawn with care. The questionnaire consisted of self-report measures, which are sensitive to social desirability bias, and items were not validated as they were used merely as control variables in this study. Future studies on verbal prompts may, however, benefit from validating a perceived-obtrusiveness scale, such as the one developed by van Kleef et al. [25] and measuring consumption of the target products using an objective measure, such as the weight of leftovers.

During the training of the telephone operators, the field study was described as the evaluation of a new type of telephone script and telephone operators were asked not to talk to each other about the experiment. However, the telephone operators were aware of recent attempts of the hospital to increase protein intake among patients and the target products were known as protein-rich products. Telephone operators were thus not fully blind to our hypotheses, which is a third limitation of this study. Efforts were made to minimize this limitation by encouraging telephone operators to strictly adhere to the telephone script in all test condition and results showed that telephone operators did not differ in terms of friendliness or helpfulness in the different conditions.

Although this study focused on increasing protein content of food orders, verbal prompts could also be used to increase consumption in general. In this field study, patients’ mean daily caloric intake was 1712 kcal and thus well below the recommended daily caloric intake of 2000 kcal for women and 2500 kcal for men. To increase caloric intake among patients, hospitals could use a verbal prompt to encourage patients to order snacks between their main meals or use a verbal prompt at every main meal rather than only for lunch. However, future research would have to examine the effectiveness of verbal prompts when consumers are repeatedly presented with them.

## Conclusions

This study demonstrated that simple and low-cost marketing techniques can have substantial effects on food choice in a natural setting, such as a hospital, and that verbal prompts are a promising type of marketing technique in this respect. Although motivating patients to change their eating habits for a longer period of time is challenging, verbal prompts may be a useful tool for health professionals to stimulate healthy food consumption among patients at least during hospitalization. On a longer term, motivating patients to explore foods they otherwise would not have tried might inspire them to start using these foods on a more regular basis [44]. However, respondents to the questionnaire indicated that the taste of the target product was their most important reason to both *order* and *not order* the target product. Foods that are generally seen as tasty may thus be most suitable as targets for verbal prompts.
